# Mobile genetic element proliferation and gene inactivation impact over the genome structure and metabolic capabilities of *Sodalis glossinidius*, the secondary endosymbiont of tsetse flies

**DOI:** 10.1186/1471-2164-11-449

**Published:** 2010-07-22

**Authors:** Eugeni Belda, Andrés Moya, Stephen Bentley, Francisco J Silva

**Affiliations:** 1 Institut Cavanilles de Biodiversitat i Biologia Evolutiva, Universitat de València. Apartat 22085, València E-46071, Spain; 2 CIBER en Epidemiología y Salud Pública (CIBEResp), Barcelona, Spain; 3 Unidad Mixta de Investigación de Genómica y Salud (Centro Superior de Investigación en Salud Pública, CSISP/Institut Cavanilles, Universitat de València, Spain; 4 Pathogen Sequencing Unit, Sanger Institute, Hinxton, UK

## Abstract

**Background:**

Genome reduction is a common evolutionary process in symbiotic and pathogenic bacteria. This process has been extensively characterized in bacterial endosymbionts of insects, where primary mutualistic bacteria represent the most extreme cases of genome reduction consequence of a massive process of gene inactivation and loss during their evolution from free-living ancestors. *Sodalis glossinidius*, the secondary endosymbiont of tsetse flies, contains one of the few complete genomes of bacteria at the very beginning of the symbiotic association, allowing to evaluate the relative impact of mobile genetic element proliferation and gene inactivation over the structure and functional capabilities of this bacterial endosymbiont during the transition to a host dependent lifestyle.

**Results:**

A detailed characterization of mobile genetic elements and pseudogenes reveals a massive presence of different types of prophage elements together with five different families of IS elements that have proliferated across the genome of *Sodalis glossinidius *at different levels. In addition, a detailed survey of intergenic regions allowed the characterization of 1501 pseudogenes, a much higher number than the 972 pseudogenes described in the original annotation. Pseudogene structure reveals a minor impact of mobile genetic element proliferation in the process of gene inactivation, with most of pseudogenes originated by multiple frameshift mutations and premature stop codons. The comparison of metabolic profiles of *Sodalis glossinidius *and tsetse fly primary endosymbiont *Wiglesworthia glossinidia *based on their whole gene and pseudogene repertoires revealed a novel case of pathway inactivation, the arginine biosynthesis, in *Sodalis glossinidius *together with a possible case of metabolic complementation with *Wigglesworthia glossinidia *for thiamine biosynthesis.

**Conclusions:**

The complete re-analysis of the genome sequence of *Sodalis glossinidius *reveals novel insights in the evolutionary transition from a free-living ancestor to a host-dependent lifestyle, with a massive proliferation of mobile genetic elements mainly of phage origin although with minor impact in the process of gene inactivation that is taking place in this bacterial genome. The metabolic analysis of the whole endosymbiotic consortia of tsetse flies have revealed a possible phenomenon of metabolic complementation between primary and secondary endosymbionts that can contribute to explain the co-existence of both bacterial endosymbionts in the context of the tsetse host.

## Background

Symbiotic associations between bacteria and insects are widespread in nature, being postulated as one of the key factors of their evolutionary success. Bacterial endosymbionts allow insects to colonize novel ecological niches characterized by unbalanced nutritional sources which would be unavailable without their assistance [1,2]. Based on the evolutionary age of the symbiotic association and the extent of codependence between both symbiotic partners, bacterial endosymbionts are classified into primary and secondary. The former are generally essential for their hosts, reside exclusively inside specialized host cells called bacteriocytes, are transmitted by strict vertical transmission from mother to descendents and the associations with their insect hosts are usually ancient. On the other hand, secondary endosymbionts have been more recently acquired by their insect hosts, they are not strictly necessary for host survival, possess a wider tissue tropism, being found both intracellularly and extracellularly in different host tissues and, although mainly transmitted by vertical inheritance, may occasionally be transmitted horizontally, even between species [3-5]. Evolutionary symbiotic associations between bacteria and insects, especially when they become obligate intracellular mutualists, have led to several common genomic features [3]. Many nonessential genes are inactivated and their DNA is lost in a stepwise process including many small and some large indel mutations [6-9]. The gradual process leads to very small genome sizes, such as those of the aphid endosymbiont *Buchnera aphidicola *[4-8], the ant endosymbiont *Blochmannia *[9,10], or the psyllid endosymbiont *Carsonella rudii *[11,12]. Drastically reduced genomes contain gene repertoires with very few, if any, presence of mobile genetic elements and encode highly streamlined metabolisms. Sequence evolution is especially distinctive with fast nucleotide substitution rates, specially for nonsynonymous substitutions [13], a strong nucleotide compositional bias toward the increase in A+T, a drastic change in the amino acid composition of proteins and an almost complete loss of the bias of codon usage, which may discriminate between high and low expressing genes [14,15]. Gene order stability is another important characteristic of many of these endosymbionts, because almost none genome rearrangement or horizontal gene transfer event is detected [5,10,16,17]. The absence of genome flux from other sources may be due to both the restricted intracellular environment and the inability to incorporate foreign DNA by recombination [18]. The strict vertical transmission from mothers to descendents leads to the coevolution between the bacterial endosymbionts and their hosts [19-21].

By contrast, the genomes of facultative secondary endosymbionts that have established recent associations with their hosts show heterogeneous genomic features. In some cases, such as sequence evolution, they show intermediate characteristics between free living and obligate endosymbionts [22,23], while in other cases they already maintain some typical characteristics of free-living species. In recent associations, genome sizes are similar to those of free-living relatives, with very few, if any, compositional bias, higher loads of mobile genetic elements (insertion sequences, transposons, prophages), and an increased amount of non-functional DNA, allowing to study the main evolutionary forces acting during this initial stages of the genome reduction process.

*Sodalis glossinidius *is the facultative secondary endosymbiont of the tsetse fly (Diptera: Glossinidae), where it is harboured both intracellularly and extracellularly in different tissues including the midgut, haemolymph, fat body, or milk-gland [24-26]. Tsetse flies harbour other two bacterial endosymbionts, the obligatory mutualist *Wigglesworthia glossinidia*, that resides intracellularly inside bacteriocytes, cells that forms the bacteriome organ in the anterior midgut [27,28], and the parasitic *Wolbachia*, that is localized in ovarian tissues [29].

The genome of *W. glossinidia *was sequenced in 2002 [30], and shows the typical features of an ancient obligatory primary endosymbiont, with a highly streamlined chromosome (698 kb) with 22% GC content and 621 protein coding genes (CDSs). It has retained most genes for cofactor biosynthesis, which is claimed as the main reason for this symbiotic association since endosymbionts may supply the tsetse host with vitamins, which are absent in the vertebrate blood. By contrast, the genome sequence of *S. glossinidius *[31] revealed genomic features closer to free-living γ-proteobacteria like *Escherichia coli *than to obligatory mutualists, with a genome size of 4.17 Mb and 54.7% GC content. Its main feature was the detection that close to one fourth of CDSs were inactive (972 pseudogenes), indicative of a massive process of gene inactivation, which has also been described in other bacterial association, such as the one of the pathogenic bacteria *Mycobacterium leprae *[32,33]. The absence of coevolution between *S. glossinidius *and tsetse flies and the fact that *S. glossinidius *is the only bacterial endosymbiont that is able to be cultured *in vitro *reveals a very recent symbiotic association with the tsetse host [34-36], providing an ideal system to study the initial steps of the transition from free-living to a host-dependent lifestyle.

We present a complete re-annotation and re-analysis of the genome of *S. glossinidius str. morsitans *centered in three main aspects. First, the detailed characterization of pseudogenes at both nucleotide and amino acid levels, second, characterization of the different types of insertion sequence (IS) and prophage elements present in the genome and the assessment of their role in the gene inactivation process, and third, the detailed analysis of the metabolic system comprised by *S. glossinidius *and *W. glossinidia *in order to evaluate the impact of gene inactivation over metabolic capabilities of *S. glossinidius *and the extent of metabolic complementation between both symbionts in the context of the symbiotic association with the tsetse flies.

## Methods

### Pseudogene annotation

The genome and coding sequences of *S. glossinidius *(strain Morsitans) were extracted from GenBank [GenBank: NC_007712]. Pseudogene boundaries were characterized by a two-step approach. In a first step, intergenic regions between non-overlapping genes with a minimum length of 50 base pairs (2215 intergenic regions) were used as query sequences against *Escherichia coli *strain K12 substrain MG1655 proteome [GenBank: NC_000913] using BLASTX [37] with a maximum e-value cutoff of 10^-5^, extracting the segments covering each BLASTX hit in *S. glossinidius *genome. Potential pseudogenes covering at least 25% of encoded protein length were retained, rendering a preliminary set of 845 potential pseudogenes. In a second step, new intergenic regions were extracted this time considering originally annotated genes and previously identified pseudogenes and those with a minimum length of 50 base pairs (2378 intergenic regions) were used as query sequences against proteomes of all completely sequenced bacterial genomes in KEGG database [38] at December of 2007 using BLASTX with a maximum e-value cutoff of 10^-5^. This analysis produced a second preliminary set of 879 potential pseudogenes. Finally, Genewise program [39] was used to predict the open reading frames of each potential pseudogene based on the protein sequence of their best BLASTX hit, and this information was integrated with Artemis software release 10 [40] joining open reading frames of the same pseudogene.

### Insertion sequence element characterization

To characterize the major types of IS elements of *S. glossinidius*, its genome sequence was self-compared using NUCMER program from MUMMER package [41] with default conditions. Four main blocks of highly conserved repeated sequences with a minimum sequence length of 500 bp were identified. They included 28 out of 29 originally annotated transposase genes. In order to cover the complete IS length, repeated sequences of each type (named ISSgl1, ISSgl2, ISSgl3 and ISSgl4) plus 100 bp flanking regions were extracted (16, 6, 2 and 4, respectively). Sequences were aligned with ClustalW program [42]. Inverted repeats were identified with the programs *Palindrome *and *Etandem *included in the EMBOSS package [43]. Direct repeats flanking each IS element were identified by visual inspection of nucleotide sequences. Consensus sequences for each alignment were extracted with *Consense *program included in the EMBOSS package. More divergent or partial copies of each IS element were identified using the consensus sequences as queries in BLASTN searches against *S. glossinidius *genome. Finally, in order to characterize minor IS elements not detected by the previous approach, the remaining genome sequences were used as query sequence against IS finder database [44] using BLASTX with an e-value cutoff of 10^-5^, detecting a fifth group of IS elements (named ISSgl5) that was characterized by the same procedure described above.

Genes disrupted by IS element insertion events were identified by BLASTX using the two sequences flanking each IS element as queries against non-redundant protein database subdivision of GenBank [45] with an e-value cutoff of 10 ^-5^.

For each type of IS element, complete and partial IS copies were aligned with ClustalW. The p-distance between copies of the same type of element (number of differences/alignment length) were estimated with the pairwise deletion option in MEGA4 software package [46], and the segment of the multiple alignment corresponding to each transposase gene was extracted and translated. Elements with stop codons or frameshifts in the region corresponding to the transposase gene were considered as defective, whereas, in absence of any further information, substitutions rendering amino acid changes or small indels multiples of three nucleotides were considered to yield functional products.

#### Whole genome functional reannotation

Manual re-annotation of all originally annotated protein coding genes and potential pseudogenes was carried out integrating different sources of information. Hierarchical functional classification scheme adopted from the Sanger Institute, that is derived from the more general Multifun classification scheme of gene products [47], was used to assign a "class" qualifier to each CDS reflecting their cellular function. The annotation of *E. coli *K12 genome was adopted as reference given its close evolutionary relationship with *S. glossinidius *and the precision of its annotation, recently updated by an international consortium [48]. Orthology between *S. glossinidius *and *E. coli *K12 protein coding genes was proposed based on reciprocal-best-match FASTA searches [49] with cutoffs of 80% of alignment length and 30% of amino acid identity. The annotation from *E. coli *K12 was transferred to the putative *S. glossinidius *ortholog including qualifiers "product", "EC number", "class", "gene" "function" and "note". Reciprocal-best-match FASTA searches were also carried out between *S. glossinidius *genome and HAMAP database of manually annotated microbial proteomes [50] with the same cutoffs. Annotations from HAMAP hits were transferred to *S. glossinidius *CDSs including qualifiers "primary_name", "product" and "EC_number" when they were available. The same approach was followed in FASTA searches against internal database of 24 bacterial genomes annotated by the same procedure. Finally, individual BLASTP and FASTA searches were carried out with all *S. glossinidius *CDSs against bacterial subdivision of Uniprot database [51] and additional functional data were provided by individual searches of each *S. glossinidius *CDS against protein domain databases such as PFAM and Prosite [52]. TMHMM [53], SIGNALP [54] and PSORT [55] programs were used to predict transmembrane domains, signal peptides, and cellular localization of gene products, respectively. The results of all these analyses were integrated together using Artemis software release 10 and were manually curated at both functional and physical level. The re-annotated genome sequence of *S. glossinidius *is shown as supplemental material (see Additional File 1).

#### Metabolic reconstruction

KEGG pathway maps were generated with KEGG Automatic Annotation Server [56] from amino acid sequences of genes and pseudogenes. Predicted EC numbers were compared to those transferred during functional re-annotation process in order to ensure a correct assignment of enzymatic functions. BLAST2GO program [57] and specific BLASTP searches of dubious enzymatic activities against specific entries of non-redundant protein database subdivision of GenBank, to avoid functional over-assignments, were used to complement KAAS pathway reconstruction in order to predict mutifunctional enzymes associated with more than one EC number that were not identified with KAAS. Extensive literature searches and specialized metabolic databases like ECOCYC [58] and METACYC [59] were also used during the analysis of the reconstructed metabolic pathways.

#### Whole genome comparisons

TBLASTX genome comparisons were generated between *S. glossinidius *and *E. coli *K12 genomes to help the reannotation process and metabolic reconstruction. TBLASTX comparisons were also generated between the genome of *S. glossinidius *and the complete phage genome subdivision of GenBank in order to characterize complete or partial prophage insertions. Whole genome comparisons were analyzed with Artemis Comparison Tool software version 7 [40].

## Results

### Pseudogene number adjustment

It was previously described that the genome of *S. glossinidius *harboured 972 pseudogenes [31]. However, their coordinates and the function of the active genes from which they derived were not annotated either in the manuscript or in the GenBank file. We performed a complete pseudogene re-annotation identifying and characterizing 1501 pseudogenes (Additional File 1). First, we performed a BLASTX search over *S. glossinidius *intergenic regions, which allowed us to identify 1724 potential pseudogenes. Second, individual inspection of FASTA and BLASTP results for each CDS allowed us the detection of adjacent pseudogenes that were different frames of a same original functional gene. We merged these adjacent pseudogenes in single CDSs. Third, IS element characterization allowed the detection of ancestral genes that were inactivated by their insertions, with the two parts of the ancestral gene flanked by the annotated IS element. The two segments of each disrupted gene were merged in a single CDS. Fourth, during the re-annotation process we detected 142 situations in which a putative functional gene had been split in an ORF included in the primary annotation as a functional gene and a pseudogene detected in our FASTA and BLASTP searches. These pseudogenes were eliminated from the final re-annotation file and a "misc_feature" qualifier were added to the corresponding functional genes specifying the proportion of the ancestral gene represented by the originally annotated gene and the characterized pseudogene (see Additional File 2). Finally, potential pseudogenes with no significant homology on BLASTP and FASTA searches and no possible functional inference by domain analysis (PFAM or Prosite) or cellular location (TMHMM or PSORT analysis) were eliminated. Despite the reduction over the initial estimations, the final set of 1501 pseudogenes overcame the 972 pseudogenes described in the original annotation [31].

### Whole genome functional re-annotation

**Figure 1:**
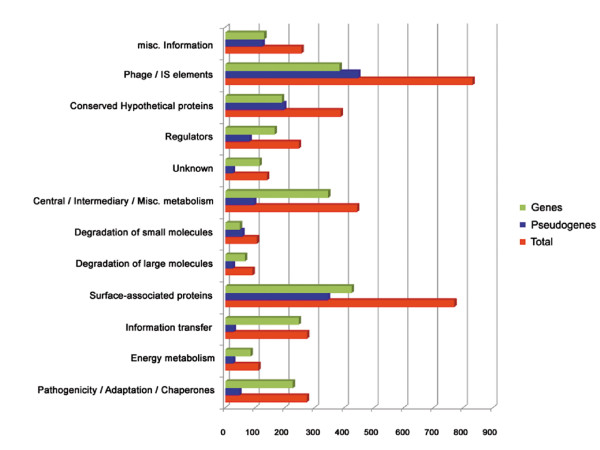
**Results of the re-annotation process**. The functional categories correspond to the "colour" qualifier in Additional File 1. Red bars show the number of CDSs (genes and pseudogenes) in each functional category. Green bars show the number of genes in each functional category. Blue bars show the number of pseudogenes in each functional category.

A systematic re-annotation of all CDSs was performed based on information derived from similarity searches (BLASTP, FASTA), protein motif searches (PFAM, PROSITE, TMHMM and SIGNALP), and protein localization prediction (PSORT). Using the functional classification scheme of the Sanger Institute (Additional File 3), we classified CDSs in functional categories (Figure 1). The highest gene proportion was assigned to surface protein (770 CDSs) and mobile genetic element (831 CDSs) functional classes. These numbers correspond to 19.6% and 21.1% of the total number of CDSs, respectively. Surface protein functional class includes all integral membrane proteins (inner and outer membrane proteins), secreted proteins, and membrane transporters (ABC transporters, PTS system components, etc), but also all genes encoding for enzymatic activities involved in peptidoglycan and bacterial lipopolysaccharide biosynthesis. Mobile genetic element class contains all transposase coding genes and all genes encoding proteins with homology with prophage elements, including integrases, reverse transcriptases, phage tail proteins and hypothetical phage proteins. In addition, these two functional classes harbour the highest number of pseudogenes with 345 and 447 pseudogenes respectively, being also the functional classes most affected by pseudogenization, with pseudogenes representing 44.8% and 53.8% of the CDSs assigned to surface and mobile genetic elements classes, respectively.

**Figure 2:**
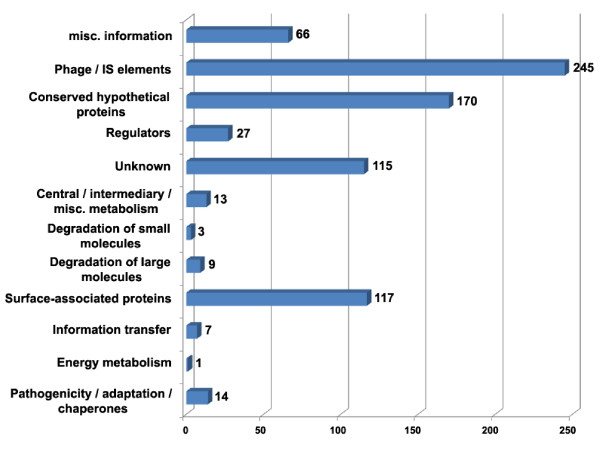
**Functional re-assignments of genes originally annotated as "hypothetical proteins"**. Functional categories correspond to the "colour" qualifier in the final re-annotation file. There is a single gene re-assigned to "energy metabolism" category.

The original annotation described 787 genes encoding hypothetical proteins. After the re-annotation process 190 remained annotated as conserved hypothetical proteins, which means that the corresponding proteins are conserved in several bacteria but have an unknown function, and 115 as unknown proteins, representing orphan genes with the corresponding protein having no significant homology with any entry of public databases. This latter result contrasts with the 221 genes described in the original *S. glossinidius *genome paper with no homology with any entry from public databases [31]. This is a consequence of the exponential growth in the number of complete bacterial genomes due to the new generation of sequencing technologies (454, Solexa, and SOLID sequencing techniques), with more than 1,000 complete bacterial and archaeal genomes available since the publication of the genome of *S. glossinidius *in January, 2006 (http://www.genomesonline.org). In addition, the use of different sources of information out of BLASTP and FASTA searches allowed to make functional inferences for proteins with no clear functional assignment based only on sequence similarity searches. A detailed survey of functional reassignments of the 787 protein coding genes originally annotated as hypothetical proteins indicates that most functional reassignments were done to mobile DNA (245 genes) and to surface protein (117 genes) functional classes (Figure 2).

The classes with the fewest number of CDSs are those corresponding to enzymes for degradation of large (91 CDSs) and small (106 CDSs) molecules, representing 2.3% and 2.7% of the total number of CDSs, respectively (Figure 1). It agrees with the previous observation that *S. glossinidius *has mainly retained biosynthetic pathways [31].

**Figure 3:**
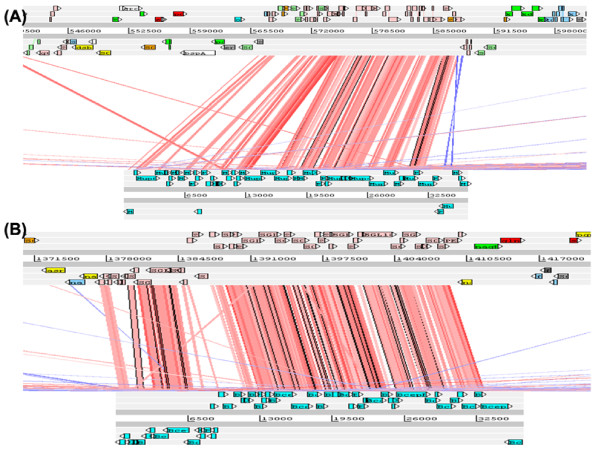
**Complete prophages characterized during the re-annotation process. **Comparisons were generated with ACT based on TBLASTX comparisons of whole genome sequences: (a) *S. glossinidius *complete prophage region SGLp1 (top) vs. enterobacteria phage Mu (NC_000929.1) (bottom) (b) *S. glossinidius *complete prophage region SGLp2 (top) vs. Burkholderia phage BcepMu (NC_005882) (bottom).

Due to the massive presence of phage-related CDSs (698), a more detailed analysis was done. They represent a 17.8% of the total CDSs of the genome, being strongly affected by gene inactivation with 353 pseudogenes. These CDSs were confirmed as phage-related genes by BLASTP searches against ACLAME database of mobile genetic elements (http://aclame.ulb.ac.be/), with only 41 genes and 62 pseudogenes having no-significant homology with any phage-related gene from the database (E-value cutoff = 10^-6^) but showing significant homology with hypothetical phage proteins from non-redundant databases on BLASTX searches. TBLASTX analysis of *S. glossinidius *complete genome against all completely sequenced phage genomes in GenBank allowed the detection of two genome regions with homology with two completely sequenced bacteriophage genomes belonging to the Mu-family of double-stranded DNA bacteriophages. The first region, named SGLp1, is located between pseudogenes ps_SGL0195c and ps_SGL0213 and shows strong colinearity with enterobacterial phage Mu [Genbank: NC_000929.1] at whole genome level. Gene inactivation has affected to 19 of the 28 CDSs included in the prophage region including the genes *c *and *ner*, involved in the regulation of lysogeny and lytic development, *A *and *B *involved in phage integration and transposition, *I *encoding a protease and scaffolding protein, and the majority of phage tail assembly genes, indicating the inactivity of this prophage element [60]. In addition, there are 5 orphan genes with no homology with any protein coding gene from ACLAME database or non-redundant databases reflecting the modularity associated with phage evolution, with regions of significant homology interdispersed with unrelated segments [61,62]. The second region, named SGLp2, includes 47 CDSs comprised between SG0816 gene and pseudogene ps_SGL0453, and shows strong colinearity at whole genome level with *Burkholderia phage BcepMu *[GenBank: NC_005882], a Mu-like bacteriophage isolated from *Burkholderia cenocepacia strain J2315 *that has closely related homologs in prophage elements from several γ-proteobacterial genomes, sharing a high degree of colinearity, with much less of the mosaicism detected in other bacteriophages, including a common inversion of the entire left end region of their genomes compared with other Mu-like prophages [63] also present in the prophage region of SGLp2 (Figure 3). In contrast with SGLp1, where gene inactivation has affected the majority of CDSs of the prophage region, in SGLp2 only 5 out of 47 CDSs are pseudogenes, retaining a completely functional lysis gene cassette for cell adhesion and invasion together with functional genes for capside formation and DNA packaging, and genes involved in the biosynthesis of the contractile phage tail [64]. However, there is no homology at the level of phage control region, that is also absent in the homologous prophage regions of *S. typhi CT18 *and *S. typhi Ty2*, indicating that these prophages are uninducible cryptic prophages [64]. In addition to this two complete prophage elements, there are 11 genome regions that show significant homology with different domains of completely sequenced phage genomes including 2 domains with homology with phage epsilon 15 [GenBank: NC_004775.1], that is one of the precursors of *S. glossinidius str. Morsitans *extrachromosomal bacteriophage-like element pSOG3 [65] (Additional File 4).

### Insertion sequence characterization

Due to the massive presence of CDSs belonging to mobile genetic element functional class and the proposed role of IS element proliferation in the first steps of genome reduction of bacterial endosymbionts [66-68], a complete characterization of the different types of IS elements was carried out. Five different types of IS elements were identified in the genome of S. *glossinidius *representing 2.52% of the overall genome sequence, all of which showed clear homology with known γ-proteobacterial IS families, with their main structural characteristics described in Table 1. There are clear differences between IS types in terms of functionality and sequence divergence, which are indicative of different stages of IS degeneration. Among the five types of IS elements, only ISSgl1 and ISSgl2 can be considered as functional IS elements by the presence of functional transposase genes clearly conserved at whole sequence level between IS copies and with homology with complete PFAM domains corresponding to transposases. The most abundant IS element is ISSgl1, that belongs to IS5 family of IS sequences, with a functional transposase gene that encodes for a protein of 307 amino acids with a functional DDE domain common to most transposase genes responsible for co-ordinate divalent metal ions, mainly Mg^+2^, needed during the course of the transposition reaction [69]. Among the 16 genes originally annotated as transposase genes belonging to ISSgl1 type, only 6 contained a complete DDE domain and can be considered as functional IS elements. The other IS type with functional transposase is ISSgl2, that belongs to IS110 family of IS elements, a group characterized by the absence of inverted repeats in most of its members as well as the absence of direct repeats flanking the IS element after transposition, also observed in complete ISSgl2 elements [70]. However, inverted repeats of 11 bp are detected internally of consensus ISSgl2 sequence, with right inverted repeat located inside the transposase gene (See Additional File 5). The 6 transposase gene copies included in this IS type presented the same profile on PFAM searches, being all potentially functional transposases except in one case where we detected a premature stop codon in one of the transposase genes belonging to a truncated ISSgl2, probably reflecting an ongoing process of IS degradation. For the rest of IS types (ISSgl3, ISSgl4, ISSgl5), it was no possible the identification of a functional transposase gene common to all components of the IS type, despite each IS type included originally annotated transposase genes.

**Table 1:** Structural and evolutionary characteristics of the 5 main groups of IS elements characterized in the genome of *S. glossinidius*.

		ISSgl1	ISSgl2	ISSgl3	ISSgl4	ISSgl5
**IS family**		**IS5**	**IS110**	**IS256**	**IS110**	**ISNCY**

Structural features						
	Consensus sequence length (bp)	1052	1175	1247	1210	939
	GC content (%)	49.8	49.7	51	48.7	52.7
	Number of open reading frames	1	1	1	1	1
	Inverted repeats (bp)	17	11	34	10	n.d. ^(c)^
	Direct repeats derived from transposition (bp)	9	n.d. ^(d)^	9	n.d. ^(d)^	n.d. ^(d)^

Complete copies ^(a)^		47	9	7	7	4

Partial copies ^(b)^		16	9	12	2	9

IS with functional transposase genes		10	4	0	0	0

Pseudogenes by IS insertion		10	2	2	3	0

p-distance (number of nucleotide differences/total length)						
	Minimum	0.0	0.0024	0.0062	0.0048	0.1097
	Maximum	0.066	0.0268	0.0756	0.0240	0.3935
	Mean	0.02	0.0162	0.0287	0.0131	0.2388

Total length (nt) ^(e)^		52130	15328	16915	9375	11411

^a ^Complete copies are those that present inverted repeats at both ends of the transposase gene.

^b ^Partial copies correspond to fragments of the putative IS element.

^c ^No inverted repeats were detected in ISSgl5

^d ^No direct repeats were detected in ISSgl2, ISSgl4, and ISSgl5 (n.d.).

^e ^Sum of the length of all corresponding complete and partial IS elements.

In addition, 3 complete and 5 partial IS elements presents in single copy were characterized during the re-annotation process. This raises the fraction of *S. glossinidius *genome represented by IS elements to 2.72%.

Of the 1501 characterized pseudogenes, only 18 were originated by the insertion of an IS element indicating that IS transposition has not been a major force in the process of psudogenization in *S. glossinidius*.

### Metabolic reconstruction

**Figure 4:**
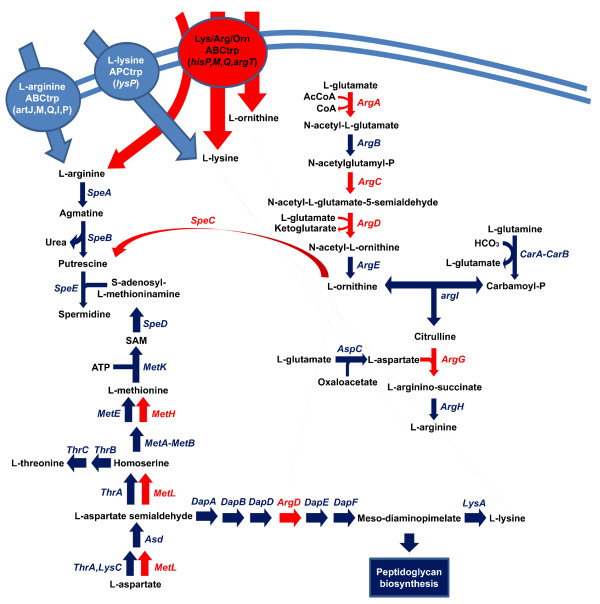
**Metabolic profile of L-arginine biosynthesis from L-glutamate in *S. glossinidius *and its implication in spermidine, L-lysine and peptidoglycan biosynthesis**. Red coloured enzymatic reactions represent gene inactivation events, whereas blue coloured ones are those encoded by functional genes.

A detailed analysis of the metabolic map of *S. glossinidius *was carried out based on the combined results of KEGG Automated Annotation Server and Blast2GO with the complete set of genes and pseudogenes (3932 CDSs) together with extensive literature searches and specialized metabolic databases like ECOCYC or METACYC that allowed to detect several features do not described in the original annotation. The most important was the inactivation of the pathway for L-arginine biosynthesis from L-glutamate (Figure 4). This pathway proceeds through eight enzymatic steps, with five steps involved in L-ornithine production from L-glutamate and three steps for the production of L-arginine from L-ornithine and carbamoyl phosphate, an essential intermediate of pyrimidine biosynthesis [71]. Two main patterns for L-arginine biosynthesis differing in the strategy followed to remove acetyl group from the intermediate N-acetyl ornithine have been described. One is a linear pathway found in members of the Enterobacteriaceae and Bacilleae in which N-acetyl ornithine is deacetylated by the hydrolytic enzyme acetylornithine deacetylase encoded by the gene *argE *producing L-ornithine and acetate. The other is a cyclic pathway, energetically more efficient and found in most prokaryote and eukaryote microbes, which involves a transacylation of N-acetyl ornithine using glutamate as acetate acceptor yielding L-ornithine and N-acetyl-L-glutamate, the first intermediate of the pathway, through a reaction catalyzed by ornithine acetyltransferase encoded by the gene *argJ *[71]. In addition, a third mechanism has been identified in Xanthomonadales in which N-acetyl-ornithine is directly transcarbamylated to N-acetyl citrulline by a specific N-acetyl ornithine transcarbamylase and then deacetylated by the common acetylornithine deacetylase (*ArgE*) to the L-arginine precursor citrulline [72]. The ancestor of *S. glossinidius*, as other members of enterobacteriaceae, presented a complete linear pathway for L-arginine biosynthesis from L-glutamate, however, in *S. glossinidius*, gene inactivation has affected the first (*argA*), third (*argC*), fourth (*argD*) and seventh (*argG*) steps of the pathway (Figure 4) indicating a complete inability for L-arginine biosynthesis by none of the above described pathways given the inactivation of three of the four enzymatic activities involved in the biosynthesis of the common intermediate N-acetyl ornithine. The inactivation of the arginine biosynthetic pathway indicates that *S. glossinidius *needs L-arginine supply from the tse-tse host, which will be accomplished by a functional arginine ABC transport system encoded by the genes *artM, artQ, artI *and *artP *[73] despite the inactivation of the major Lysine/Arginine/Ornithie (LAO) ABC transport system (*hisM *(ps_SGL0977c) and *hisQ *(ps_SGL0978)). The inactivation of the major LAO ABC transport system has also consequences in other biosynthetic pathways like putrescine biosynthesis, a polyamine involved in cell development that can be synthesized by two alternate pathways from L-arginine or L-ornithine. In concordance with the inactivation of L-ornithine biosynthesis from L-glutamate and L-ornithine transport through LAO ABC transport system, *S. glossinidius *has retained functional *speA *(encoding arginine decarboxylase) and *speB *(encoding agmatinase) genes for putrescine biosynthesis from L-arginine, but has inactivated *speC *gene (encoding ornithine decarboxylase) required for putrescine biosynthesis from L-ornithine.

The *argD *gene has also been postulated to additionally encode a succinyldiaminopimelate aminotransferase activity, required for the sixth step of L-Lysine and Mesodiaminopimelate biosynthetic pathway that produces L-Lysine and their precursor Mesodiaminopimelate, that constitutes an essential cross-linking moiety of the peptidoglycan component of bacterial cell walls [74]. However, this evidence comes from in-vitro assays of purified ArgD protein with different substrates, and shows that the affinity for acetylornithine is higher than for succinyldiaminopimelate. In addition, other studies have determined that there is a specific succinyldiaminopimelate activity in *Escherichia coli *additional to *argD *encoded aminotransferase [75], in concordance with the presence of PLP-dependent aminotransferases of unknown function in the genome of Escherichia coli like *b2290*, that encodes a predicted PLP-dependent aminotransferase. It has a functional ortholog in the genome of *Sodalis glossinidius *(SG1602) not assigned to any pathway, so probably this uncharacterized PLP-dependent aminotransferases are responsible for succinyldiaminopimelate aminotransferase activity in L-lysine biosynthetic pathway. Alternatively, it has been also described the replacement of mesodiaminopimelate by the methionine biosynthesis intermediate cistathionine in *E. coli *[76], so another possible explanation is that cystathionine replaces mesodiaminopimelate in peptidoglycan biosynthesis, leading to the dependence of extracellular L-lysine through a functional APC transporter encoded by the gene *lysP *(SG0955).

**Figure 5:**
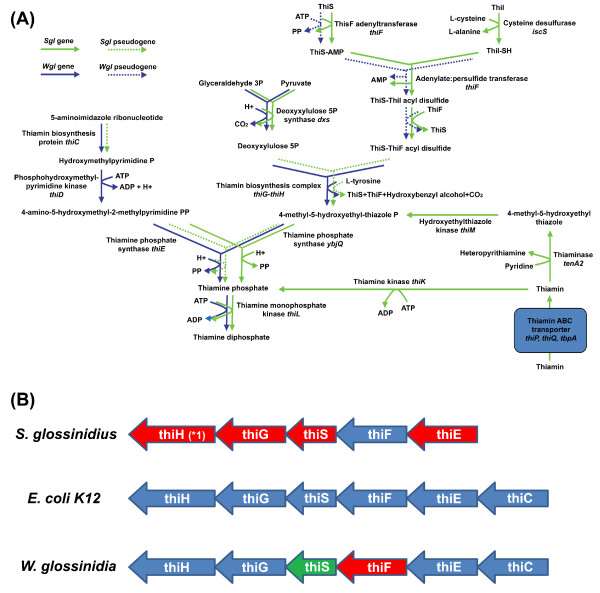
**Metabolic profile of thiamine biosynthesis pathway in *S. glossinidius *and *W. glossinidia***. (A) Schematic diagram of thiamine biosynthesis pathway. Reactions coloured in blue represents the functional profile of thiamine biosynthesis in *W. glossinidia*, whereas reactions coloured in green represents the functional profile of thiamine biosynthesis in *S. glossinidius*; Pseudogenized activities are represented by dashed lines. Functional thiamine ABC transport system, thiamine kinase (*thiK*) and thiamine phosphate kinase (*thiL*) allow thiamine diphosphate biosynthesis from exogenous thiamine. (B) Structure of the thiamine biosynthesis operon in *S. glossinidius*, *E. coli K12*, and *W. glossinidia*. Functional genes (blue), pseudogenes (red), and the *thiS *gene of *W. glossinidia *characterized in this study (green). The *thiH *gene in *S. glossinidius *corresponds to an originally annotated gene (*SG0136*) with a premature stop codon detected during the reannotation process (*1)

Another important feature of the *S. glossinidius *metabolism refers to the different pathways for cofactor biosynthesis, which have been postulated as the driving force in the maintenance of the symbiotic association between tse-tse flies and their primary endosymbiont *W. glossinidia *[25,77]. A detailed analysis of the metabolic maps of *S. glossinidius *based on KEGG Automated Annotation Server and BLAST2GO results revealed that *S. glossinidius *does not contain a complete thiamine phosphate biosynthetic pathway. This suggested that not only tsetse flies but *S. glossinidius *were dependent of *W. glossinidia *for its synthesis. However, the analysis of the thiamine biosynthetic pathway in *W. glossinidia*, although leads to the detection of some previously non annotated genes, also revealed that the pathway was incomplete lacking the essential *thiI *gene and showing *thiF *as a pseudogene. The synthesis of the biologically active cofactor thiamine diphosphate in bacteria [78-81] takes place by several pathways (Figure 5). The *de novo *pathway requires the synthesis of two intermediates, a pyrimidine phosphate moiety and a thiazole phosphate moiety. Both are combined by the action of a thiamine phosphate synthase (ThiE) and a thiamine phosphate kinase (ThiL) to produce the active cofactor.

The analysis of this metabolism in *S. glossinidius *(see genes in Table 2) showed that the ancestor of this bacterium probably contained the complete set of genes for its synthesis. However, a process of degeneration, recently started, completely avoids its *de novo *biosynthesis yet. Remnants of this situation are 4 pseudogenes (*thiS*, *thiF*, *thiG *and *thiE*) together with an originally annotated gene (*thiH*) that presents a premature stop codon probably reflecting an ongoing gene inactivation event. Thus, the most plausible explanation for the presence of the cofactor in *S. glossinidius *is a salvage pathway through the presence of a functional thiamine ABC transport system encoded by the genes *thiP*, *thiQ *and *tbpA *together with a functional thiamine kinase (ThiK) and thiamine phosphate kinase (ThiL) (Figure 5A). However, it implies that sufficient amounts of thiamine should be available from the host, something improbable. In addition, the *S. glossinidius *proteome contains remnants activities and proteins related with the pathway, including the ability to synthesize thiazole phosphate carboxylate (THZ-P) through a salvage pathway.

**Table 2:** Functional status of genes involved in thiamine diphosphate biosynthesis in *S. glossinidius *and *W. glossinidia*.

		*S. glossinidius*			*W. glossinidia*		
**Gene name**	**Enzyme**	**Gene**	**Pseudog.**	**Absent**	**Gene**	**Pseudog.**	**Absent**

***thiI***	Sufur-carrier protein ThiI	**+**					**+**

***thiF***	Thiamin (thiazole moiety) biosynthesis protein ThiF	**+**				**+**	

***iscS***	Cysteine desulfurase	**+**					**+**

***thiS***	Sulfur-carrier protein ThiS		**+**		+(**)		

***dxs***	1-deoxyxylulose-5-phosphate synthase	**+**			**+**		

***thiG***	ThiG subunit of thiamin biosynthesis complex ThiGH		**+**		**+**		

***thiH***	ThiH subunit of thiamin biosynthesis complex ThiGH		+(*)		**+**		

***thiC***	Thiamin (pyrimidine moety) biosynthesis protein ThiC		**+**		**+**		

***thiD***	Bifunctional hydroxymethylpyrimidine kinase/phosphohydroxymethylpyrimidine kinase			**+**	**+**		

***thiE***	Thiamin phosphate synthase		**+**		**+**		

***ybjQ***	Conserved protein with activity thiamin phosphate synthase	**+**					**+**

***thiL***	Thiamin-monophosphate kinase	**+**			**+**		

***thiM***	Hydroxyethylthiazole kinase	**+**					**+**

***thiK***	Thiamin kinase	**+**					**+**

***tenA2***	Thiaminase	**+**					**+**

***thiP***	Thiamin ABC transport system; fused membrane components	**+**					**+**

***thiQ***	Thiamin ABC transport system; ATP-binding protein	**+**					**+**

***tbpA***	Thiamin ABC transport system; substrate-binding protein	**+**					**+**

^(*) ^Originally annotated gene with premature stop codon

^(**) ^Gene absent in *W. glossinidia *in original annotation but detected in the present study by TBLASTN against *E. coli K12 *genome

The analysis of the metabolism in *W. glossinidia *(genes in Table 2) revealed that it retains functional *thiH*, *thiG*, *thiE*, and *thiC *genes, with no signal of *thiI *gene, and with *thiS *and *thiF *genes non annotated but present in the intergenic region between *thiG *and *thiE *(Figure 5B). TBLASTN analyses with ThiF and ThiS from *E. coli *against the complete genome of *W. glossinidia *confirmed the presence of non annotated *thiS *and *thiF *genes immediately downstream *thiG *gene. However, whereas *thiS *has no stops nor frameshifts, the putative *thiF *gene of *W. glossinidia *contains an internal stop codon that disrupts the translation of the putative ThiF protein at amino acid 165, before the essential cysteine residue at position 184 that is responsible for the disulfide linkage between ThiS and ThiF that acts as sulfur donor in thiazole phosphate moiety biosynthesis [82]. The absence of these two genes limits the metabolic capability of *W. glossinidia *to the synthesis of the thiazole moiety (4-methyl-5-(β-hydroxyethyl)thiazole-phosphate, THZ-P).

**Figure 6:**
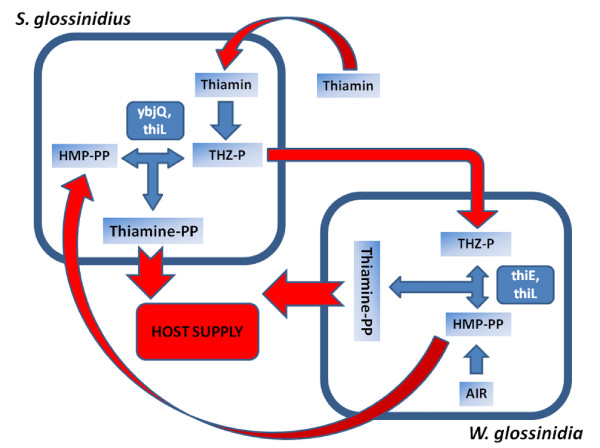
**Pontetial metabolic complementation between *W. glossinidia *and *S. glossinidius *at thiamine biosynthesis level**. Thiazole phosphate carboxylate (THZ-P) is synthesized by *S. glossinidius *from exogenous thiamin through salvage pathway (*tenA2*, *thiM*), whereas hydroxymethyl pyrimidine pyrophosphate (HMP-PP) is synthesized by *W. glossinidia *from 5-aminoimidazole ribonucleotide (AIR) (*thiC*, *thiD*). THZ-P and HMP-PP are shared between both bacteria to produce the functional thiamine diphosphate that is provided to the tsetse host.

Because the metabolic pathways of both symbionts were incomplete we searched for evidences of complementation. This was revealed through the identification of *yjbQ *gene in *S. glossinidius *(SG2130). The YjbQ protein has been recently demonstrated to contain a thiamine phosphate synthase activity able to rescue thiamine auxotrophy in a mutant *thiE *strain [83]. This leads to a scenario in which *W. glossinidia *synthesizes HMP-PP, and *S. glossinidius *THZ-P. Both intermediates may be shared by the endosymbionts with *W. glossinidia *synthesizing the active cofactor after the action of ThiE and ThiL and *S. glossinidius *after the action of YjbQ and ThiL (Figure 6).

In addition, detailed analysis of cofactor metabolism in *W. glossinidia *indicated that folate and Coenzyme A biosynthesis pathways are severely impaired due to the absence of *pab *genes (*pabA, pabB, pabC*) for the biosynthesis of the folate precursor p-aminobenzoate from chorismate, whose biosynthetic pathway from pentose phosphate pathway intermediate erythrose 4 phosphate is also absent, and the absence of *ilv *genes for the biosynthesis of Coenzyme A precursor 2,3-ketoisovalerate, a common precursor for L-valine and Coenzyme A biosynthesis. All this biosynthetic pathways are completely functional in *S. glossinidius*.

## Discussion

Bacterial endosymbionts of insects are characterized by highly reduced genomes compared to their free-living counterparts due to a massive process of gene inactivation consequence of relaxed purifying selection over high proportion of protein coding genes that are non-essential in the intracellular host-rich medium and the effect of genetic drift that allows slightly deleterious mutations to be fixed in the population due to successive population bottlenecks in the transmission of bacterial endosymbionts from mother to their descendents in a process known as Muller's ratchet [13,16,84]. This process leads to the massive accumulation of pseudogenes during initial stages of the transition from free-living to host-dependent lifestyle that will be subsequently eliminated from the genome due to the deletional bias associated to bacterial chromosomes [20,85,86]. In primary bacterial endosymbionts with long-term associations with their hosts like *Buchnera aphidicola*, *Blochmannia floridanus*, or *Wigglesworthia glossinidia *their highly reduced genomes show few, if any, pseudogenes. However, it is possible to infer pseudogene remnants in the intergenic regions between orthologous genes in comparisons with their free living relatives [87,88]. In contrast, facultative endosymbionts and pathogens that recently establish intracellular associations with their hosts have bigger genomes in which the presence of pseudogenes is more evident, like the 149 pseudogenes of *Y. pestis CO92 *[89] or 254 pseudogenes of *S. flexneri 2a str. 301 *[90], with the most extreme situation being that of *Mycobacterium leprae *with more than 1000 pseudogenes [32,33,91]. *S. glossinidius *is one of the few secondary facultative endosymbionts for which their genome has been completely sequenced, revealing an ongoing process of massive genome reduction with an unprecedented number of pseudogenes indicatives of a very recent symbiotic association with their tse-tse host, also confirmed by previous phylogenetic studies that show no-coevolution between tse-tse host and *S. glossinidius *phylogenetic trees [92]. A detailed survey of *S. glossinidius *intergenic regions based on BLASTX searches allowed to increase the number of pseudogenes from 972 in the original annotation [31] to 1501. This can be explained by the different methodologies used for pseudogene identification. In the original genome paper, pseudogenes were identified based on the results of different gene-prediction programs, considering pseudogenes all CDSs with less than half the lenght of its functional homologs in BLASTP searches [31], limiting pseudogene identification to recent inactivation events that conserve start/end positions along open reading frame. By contrast, pseudogene identification based on BLASTX searches of raw nucleotide sequences against protein databases allows to identify highly degraded pseudogenes whose open reading frames are not predicted by *ab-initio *gene prediction methods or pseudogenes originated by insertion of IS elements. Similar approaches for pseudogene identification identified at least 100 additional pseudogenes in the genomes of *E. coli/Shigella *clade [93], 188 additional pseudogenes in *Y. pestis CO92 *genome [94] or 6895 potential pseudogenes over 64 prokaryotic genomes [95]. In addition, 142 originally annotated genes are susceptible to be considered as potential pseudogenes because are situations of CDSs shorter than their database homologs, detecting the absent part of the gene as a flanking pseudogene.

The results of the functional re-annotation indicates a massive presence of genes related to mobile genetic elements, being also the functional class most affected by gene inactivation (447 out of 831 CDSs). Mobile genetic element expansion has been traditionally associated to initial stages of bacterial adaptation to host-dependent lifestyle associated to a relaxed selection over non-essential genes under nutrient-rich conditions allowing expansion of mobile genetic elements without detrimental effects to the host [20,96,97]. The characterization of the complete set of IS elements presents in the *S. glossinidius *genome revealed that IS elements represent only 2.72% of the genome with an estimated IS load of 0.031 elements per kilobase, similar to other recently host-dependent bacteria [98] although much lower than the estimate loads of IS elements for the genomes of *Sitophilus oryzae *and *Sitohilus zeamays *primary endosymbionts (*SOPE *and *SZPE *respectively), the closest relatives of *S. glossinidius *within γ-proteobacteria with a divergence time estimated in some 50-100 million years ago but with an obligaroty intracellular association in bacteriocyte cells [99,100]. BLASTN comparisons with the consensus sequence of the 5 different IS elements characterized in *S. glossinidius *against the recently described sequence of four IS elements from *SOPE *[67] indicate a common origin of ISSgl1 and ISsope1 (82% of identities at nucleotide level) whereas there is no similarity between the rest of IS elements, possibly reflecting independent acquisitions posterior to the divergence of both lineages. A detailed analysis of the IS flanking regions revealed that only 18 out of 1501 identified pseudogenes were originated by IS insertion, reflecting that IS transposition has not been a major force in the gene inactivation profile of *S. glossinidius*. The majority of pseudogenes are produced by frameshift mutations or premature stop codons, indicating that gene inactivation has been produced by multiple single gene inactivation events generating a lot of non-functional DNA that will be eliminated gradually due to the inherent mutational deletional bias associated to bacterial genomes and the lack of selective pressures for the maintenance of these non-functional regions [86,88,101].

The high amount of mobile genetic elements are consequence of a massive presence of phage related CDSs (17.7% of the total CDSs number), including 2 complete and 11 partial elements with homology with complete bacteriophage genomes. In bacterial endosymbionts, bacteriophage elements have been identified in recent symbiotic associations like bacteriophages APSE-1 and APSE-2 in the secondary endosymbiont of aphids *ca. Hamiltonella defensa *associated to the protection activity of this secondary endosymbiont killing parasitoid wasp larvae [102-104] or bacteriophage WO of parasitic *Wolbachia*, originally associated to promote cytoplasmic incompatibility on invertebrate host but recently proposed to be beneficial for the host allowing to control their bacterial loads through lytic development of WO prophage [105,106]. Two of the prophage domains are homologous to phage element epsilon 15 (NC_004775.1), which has been postulated as one of the precursors of the extrachromosomally replicating element of phage origin pSG3 from *S. glossinidius str. Morsitans *[65], possibly reflecting a common origin of extrachromosomal pSG3 and this 2 prophage regions of *S. glossinidius *genome in the tse-tse host *Glossina morsitans morsitans*. In addition, transposases from IS5 family have 75% of identity at amino acidic level with a transposase encoded by pSG3, also indicative of a flux of genetic material between extrachromosomal element pSG3 and the bacterial chromosome. It is important to consider that the structure and presence of bacteriophage-like element pSG3 is highly variable among *S. glossinidius *isolates from different tsetse species [65,107], so it would be expected to observe considerable differences in structure and content of prophage elements associated with *S. glossinidius *strains from different tse-tse species.

Finally, the analysis of *S. glossinidius *metabolism revealed a metabolic profile closer to free-living bacteria than to obligate mutualists, both in terms of energy production from different carbon sources and in terms of biosynthetic capabilities for most essential metabolites and macromolecules with the unique exception of L-arginine and thiamine. Our analyses disagree with those previously reported for amino acid biosynthesis that indicated that *S. glossinidius *was able to synthesize all amino acids except L-alanine [31], and showed that L-arginine was the only amino acid lacking a complete biosynthetic pathway.

We have also observed a putative metabolic complementation between *S. glossinidius *and *W. glossinidia *to produce the active cofactor thiamine pyrophosphate based on the gene repertoire of both bacteria. *S. glossinidius *was unable to produce thiamine [31], while *W. glossinidia *genome was described as having the potential to synthesize it [30]. The screening for the complete set of genes required for thiamine biosynthesis in *W. glossinidia *showed lack of two essential components (*thiI *and *thiF*) which would avoid the *de novo *biosynthesis of thiamine. However, a detailed revision of the proteins encoded by both endosymbiotic species revealed that each endosymbiont was able to synthesize one of the two moieties that the enzyme thiamine phosphate synthase combines in the pathway for the synthesis of the active cofactor [81]. While *W. glossinidia *has the capability of synthesizing the pyrimidine moiety (HMP-PP), *S. glossinidius *has that of the thiazole moiety (THZ-P). Considering that both bacterial symbionts were able to acquire the missing metabolite from outside, they would be able to synthesize the cofactor thiamine after the action of thiamine phosphate synthase and thiamine kinase. The genes encoding these enzymes (*thiE *and *thiL*, respectively) are present in the *W. glossinidia *genome, but only the second in that of *S. glossinidius*. We have found that *S. glossinidius *contains the gene *yjbQ *described recently as a thiamine phosphate synthase gene homolog, having an alanine in position 89 of the protein that has been demonstrated experimentally in *E. coli *that increases enzyme activity [83]. However, it is important to consider that this hypothesis is based on the unique two available genomes sequences of both tsetse endosymbionts, that unfortunately come from different tsetse host species, and as a consequence may not reflect the real metabolic scenario in each tsetse host. In addition, *W. glossinidia *is essential for host reproduction and fitness, whereas *S. glossinidius *have less detrimental effects upon their specific removal from tsetse host due to their more recent association, although it produces a marked decrease in tsetse longevity, so although this potential complementation could be possible in light of genome sequences, it appears not to play a major role in tsetse physiology [108]. In addition, whereas *W. glossinidia *is detected in all tsetse species, *S. glossinidius *is not ubiquitous and appears undetected in some *Glossina *species or in different individuals of a particular population. However, the particular lineage of *Glossina morsitans morsitans *shows high density of *S. glossinidius *in comparison with other *Glossina *species [109], with similar profiles of symbiont density variation of both *S. glossinidius *and *W. glossinidia *through its development that suggests some degree of adaptative regulation of density of both tsetse symbionts by means of *Glossina morsitans morsitans *host [110]. In order to better define the adaptative value of the possible metabolic complementation, not only at thiamine but also at folate and coenzyme A biosynthesis level, both severely impaired in *W. glossinidia*, it would be necessary to obtain additional genome sequences from both tsetse endosymbionts (like *S. glossinidius *from *Glossina brevipalpis *and *W. glossinidia *from *Glossina morsitans morsitans*) to determine if the metabolic profile observed is conserved when the complete symbiotic systems of different tsetse host are compared. Recently, an experimental assessment of the thiamine requirements of *S. glossinidius *has been carried out, demonstrating experimentally the necessity of an external source of thiamine, specially in the form of thiamine monophosphate, for *S. glossinidius *survival on cell cultures, and pointing out to their supply to both *S. glossinidius *and the tsetse host by *W. glossinidia*, although no direct experimental evidence about this last assumption is provided [111].

Just a few cases of complementation between the metabolisms of two insect endosymbionts for mutual benefit have been described. Two types of complementation may be distinguished. In one case, both endosymbionts produce different end products of the metabolism (*i.e. *amino acids). In the other case, each endosymbiont controls part of the biosynthetic pathway, and the combined effort of both is required in order to produce the final product. The case of *Sulcia muelleri *and *Baumannia cicadellinicola*, endosymbionts of the sharpshooter *Homalodisca coagulata*, illustrates very well both types of complementations [112,113]. Thus, many amino acids are synthesized by *S. muelleri *and provided to *B. cicadellinicola *and to the host, except methionine and histidine, which are synthesized by *B. cicadellinicola *and provided to the co-endosymbiont and the host. The biosynthesis of one of these amino acids, methionine, is shared between both endosymbionts with *S. muelleri *providing the intermediate homoserine to *B. cicadellinicola*.

Another case of by biosynthetic pathway sharing is the tryptophan biosynthesis by the two endosymbionts of the aphid *Cinara cedri *[114]. *Buchnera aphidicola *performs the first enzyme step producing anthranilic acid. It is uptaken by the co-endosymbiont *Serratia symbiotica *which has the genes that encode the remnant steps of the pathway. Then, the produced tryptophan may be supplied to the co-endosymbiont and to the insect host.

## Conclusions

A complete re-analysis of the genome sequence of *S. glossinidius *has revealed novel insights in the initial stages of the transition from free-living to a host dependent lifestyle in this bacterial genome. The relaxed selective pressures over non-essential genome regions in the more stable environment of the tsetse host have lead to a massive proliferation of mobile genetic elements in the form of prophages and IS elements, although their impact in the process of gene inactivation is minimal, with most of the pseudogenes generated by frameshift mutations or premature stop codons. A detailed survey of intergenic regions led to the characterization a higher number of pseudogenes than in the original annotation, pointing out to the importance of sequence analysis in the characterization of highly degraded pseudogenes that is not possible to identify by "ab-initio" gene prediction methods. A detailed survey of the metabolic capabilities of genes and pseudogenes together with a comparison with the metabolic profile of tsetse fly primary endosymbiont *W. glossinidia *based on the gene repertoire of their available genome sequences revealed novel inactive pathways consequence of the gene inactivation process previously undescribed in *S. glossinidius*, like arginine biosynthesis pathway, together with a possible phenomenon of metabolic complementation between both tsetse endosymbionts at thiamine biosynthesis level. This possible metabolic complementation together with the incomplete pathways for Coenzyme A and Folate cofactors in *W. glossinidia*, both completely functional in *S. glossinidius*, shed some light about the co-existence of both bacterial endosymbionts in the context of the tsetse host.

## Abbreviations

*S. glossinidius*: *Sodalis glossinidius*; *W. glossinidia*: *Wigglesworthia glossinidia*; IS: Insertion Sequences.

## Authors' contributions

FS and AM carried out the design of the study. EB and FS carried out pseudogene annotation, characterization of IS elements and prophages, functional re-annotation and metabolic reconstructions. SB participated in pseudogene characterization and functional re-annotation. EB and FS write the manuscript. All authors read and approved the final manuscript.

## Supplementary Material

Additional file 1 **Results of the functional re-annotation process in EMBL format**. "locus_tag" qualifier were assigned differentially for genes (original annotation) and pseudogenes (ps_SGL0001-ps_SGL1501). Additional qualifiers incorporated to each CDS to improve functional assignment were "EC_number", "GO", "class" and "colour" following functional classification scheme of Additional File 2 and Figure 1 respectively, "db_xref" (interpro/UniProt/TrEMBL domains), "note", "orthologs" (based on FASTA searches), "primary_name", "product", "pseudo" (assigned to the 1501 pseudogenes) and "status". The file can be directly viewed with Artemis software release 10 (Carver et-al 2008). Homology with PFAM domains for each CDS (genes and pseudogenes) are also incorporated as additional "misc_feature" key. "repeat_unit" key represents the coordinates of the different families of IS elements characterized. Click here for file

Additional file 2 **Putative CDSs including an originally annotated gene and a re-annotated pseudogene**. The coordinates corresponds to the combined limits of the gene and pseudogene corresponding to each putative CDS. "Coverage" columns show the percentage of the putative CDS represented by the originally annotated gene and the adjacent pseudogene together with the name of the originally annotated gene. "Relative position" columns represents the relative position of gene and pseudogene in the putative CDS (5P representing the 5' end of the putative CDS and 3P representing the 3' end of the putative CDS). Putative CDS with coordinates 2812192-2814586 includes one gene and two pseudogenes. Click here for file

Additional file 3 **Functional classification outline**: Scheme of the functional classification used during the re-annotation process represented by the "class" qualifier in the Additional File 1. Click here for file

Additional file 4 ***S. glossinidius *genome regions corresponding to domains of completely sequenced phage genomes**. The coordinates are extracted from whole genome TBLASTX comparisons between *S. glossinidius *and completely sequenced phage genomes presents in GenBank at May 2008. Length, GC content, total number of CDS, genes and pseudogenes, and the best homologous phage in TBLASTX searches are represented. Click here for file

Additional file 5 **Schematic representation of the 5 main types of IS elements of *S. glossinidius***. The position of the putative transposase gene is indicated in those IS types for which a functional transposase gene has been confirmed (ISSgl1 and ISSgl2). For ISSgl2 type, the relative position of internal inverted repeats is also indicated. Click here for file
