# Supplementary material for: Mobile genetic element proliferation and gene inactivation impact over the genome structure and metabolic capabilities of Sodalis glossinidius, the secondary endosymbiont of tsetse flies
Source: BMC Genomics. 2010 Jul 22;11:449. doi: 10.1186/1471-2164-11-449 (PMC3091646; doi:10.1186/1471-2164-11-449)
Supplement: Additional file 3 — Functional classification outline: Scheme of the functional classification used during the re-annotation process represented by the "class" qualifier in the Additional File 1. [file 1471-2164-11-449-S3.DOC]

**FUNCTIONAL CLASSIFICATION OUTLINE**

**0.0.0 Unknown function, no known homologues**
0.0.2 Conserved hypothetical

**1.0.0 Cell processes**
1.1.1 Chemotaxis and mobility
1.3.1 Chaperones

**1.4.0 Protection responses**
1.4.1 Cell killing
1.4.2 Detoxification
1.4.3 Drug/analogue sensitivity

**1.5.0 Transport/binding proteins**
1.5.1 Amino acids and amines
1.5.2 Cations
1.5.3 Carbohydrates, organic acids and alcohols; PTS
1.5.4 Anions
1.5.5 Other

**1.6.0 Adaptation**
1.6.1 Adaptations, atypical conditions
1.6.2 Osmotic adaptation
1.6.3 Fe storage

**1.7.1 Cell division**

**2.0.0 Macromolecule metabolism** **2.1.0 Macromolecule degradation**
2.1.1 Degradation of DNA
2.1.2 Degradation of RNA
2.1.3 Degradation of polysaccharides
2.1.4 Degradation of proteins, peptides, glycoproteins

**2.2.0 Macromolecule synthesis, modification**
2.2.1 Amino acyl tRNA synthesis; tRNA modification
2.2.3 DNA - replication, repair, restriction/modification
2.2.5 Lipopolysaccharide
2.2.6 Lipoprotein
2.2.7 Phospholipids
2.2.8 Polysaccharides - (cytoplasmic)
2.2.9 Protein modification
2.2.10 Proteins - translation and modification
2.2.11 RNA synthesis, modification, DNA transcription

**3.0.0 Metabolism**
**3.1.0 Amino acid biosynthesis**
3.1.1 Alanine
3.1.2 Arginine
3.1.3 Asparagine
3.1.4 Aspartate
3.1.5 Chorismate
3.1.6 Cysteine
3.1.7 Glutamate
3.1.8 Glutamine
3.1.9 Glycine
3.1.10 Histidine
3.1.11 Isoleucine
3.1.12 Leucine
3.1.13 Lysine
3.1.14 Methionine
3.1.15 Phenylalanine
3.1.16 Proline
3.1.17 Serine
3.1.18 Threonine
3.1.19 Tryptophan
3.1.20 Tyrosine
3.1.21 Valine

**3.2.0 Biosynthesis of cofactors, carriers**
3.2.1 Acyl carrier protein (ACP)
3.2.2 Biotin
3.2.3 Cobalamin
3.2.5 Folic acid
3.2.6 Heme, porphyrin
3.2.7 Lipoate
3.2.8 Menaquinone, ubiquinone
3.2.9 Molybdopterin
3.2.10 Pantothenate
3.2.11 Pyridine nucleotide
3.2.12 Pyridoxine
3.2.13 Riboflavin
3.2.14 Thiamin
3.2.15 Thioredoxin, glutaredoxin, glutathione
3.2.16 Biotin carboxyl carrier protein (BCCP)
3.2.18 Isoprenoid

**3.3.0 Central intermediary metabolism**
3.3.1 2'-Deoxyribonucleotide metabolism
3.3.2 Amino sugars
3.3.3 Entner-Douderoff
3.3.4 Gluconeogenesis
3.3.7 Misc. glucose metabolism
3.3.8 Misc. glycerol metabolism
3.3.9 Non-oxidative branch, pentose phosphate pathway
3.3.10 Nucleotide hydrolysis
3.3.11 Nucleotide interconversions
3.3.13 Phosphorus compounds
3.3.14 Polyamine biosynthesis
3.3.15 Pool, multipurpose conversions of intermediate metabolism
3.3.16 S-adenosyl methionine
3.3.17 Salvage of nucleosides and nucleotides
3.3.18 Sugar-nucleotide biosynthesis, conversions
3.3.19 Sulfur metabolism
3.3.20 Amino acids
3.3.21 Other
3.3.22 Nitrogen metabolism (urease)

**3.4.0 Degradation of small molecules**
3.4.1 Amines
3.4.2 Amino acids
3.4.3 Carbon compounds
3.4.4 Fatty acids
3.4.5 Other

**3.5.0 Energy metabolism, carbon**
3.5.1 Aerobic respiration
3.5.2 Anaerobic respiration
3.5.3 Electron transport
3.5.4 Fermentation
3.5.5 Glycolysis
3.5.6 Oxidative branch, pentose pathway
3.5.7 Pyruvate dehydrogenase
3.5.8 TCA cycle
3.5.9 ATP-proton motive force

**3.6.0 Fatty acid biosynthesis**
3.6.1 Fatty acid and phosphatidic acid biosynthesis

**3.7.0 Nucleotide biosynthesis**
3.7.1 Purine ribonucleotide biosynthesis
3.7.2 Pyrimidine ribonucleotide biosynthesis

**4.0.0 Cell envelope**
4.1.0 Membrane/exported/lipoproteins
4.1.1 Inner membrane
4.1.2 Murein sacculus, petidoglycan
4.1.3 Outer membrane
4.1.4 Surface polysaccharide & antigens, EPS
4.1.5 Surface structures

**4.2.0 Ribosome constituents**
4.2.2 Ribosomal proteins - synthesis, modification
4.2.3 Ribosomes - maturation and modification

**5.0.0 Extrachromosomal**
**5.1.0 Laterally acquirred elements**
5.1.1 Colicin-related functions
5.1.2 Phage-related functions and prophages
5.1.4 Transposon-related functions
5.1.5 Pathogenicity island-related functions

**6.0.0 Regulation**
**6.1.0 Two component system**
6.1.1 Sensor kinase
6.1.2 Response regulator

**6.2.0 RNA polymerase core enzyme binding**
6.2.1 sigma factor
6.2.2 anti sigma factor
6.2.3 anti sigma factor antagonist

**6.3.0 Defined families**
6.3.1 AsnC
6.3.2 AraC
6.3.3 GntR
6.3.4 IclR
6.3.5 LacI
6.3.6 LysR
6.3.7 MarR
6.3.8 TetR
6.3.9 ROK
6.3.10 DeoR
6.3.11 LuxR (GerR)
6.3.12 MerR
6.3.13 ArsR
6.3.14 PadR

**6.5.0 Others**
6.5.1 Global regulatory functions

**7.0.0 Not classified (included putative assignments)**
